# Diagnostic value of *WIF1* methylation for colorectal cancer: a meta-analysis

**DOI:** 10.18632/oncotarget.23870

**Published:** 2018-01-03

**Authors:** Haochang Hu, Bin Li, Cong Zhou, Xiuru Ying, Min Chen, Tianyi Huang, Yuehong Chen, Huihui Ji, Ranran Pan, Tiangong Wang, Danjie Jiang, Yanfei Chen, Yong Yang, Shiwei Duan

**Affiliations:** ^1^ Medical Genetics Center, School of Medicine, Ningbo University, Ningbo, Zhejiang, China

**Keywords:** DNA methylation, colorectal cancer, WIF1, diagnostic value, meta-analysis

## Abstract

As a common antagonist of Wnt/β-catenin signaling, Wnt inhibitory factor 1 (*WIF1*) plays an important role in the tumor progression. The aim of our meta-analysis was to summarize the diagnostic value of *WIF1* methylation in colorectal cancer (CRC). Eligible studies were retrieved by a systemic search among PubMed, Embase, CNKI, and Wanfang literature databases. The diagnostic value of *WIF1* methylation for CRC was assessed by the summary receiver operating characteristics (SROC) test. Our meta-analysis of 12 studies between 1420 CRC samples and 946 control samples showed that *WIF1* hypermethylation was significantly associated with CRC (*P* < 0.001, OR = 30.10, 95% CI = 19.48-46.50). *WIF1* hypermethylation, as a diagnostic biomarker for CRC, has a pooled sensitivity of 0.40 (95% CI: 0.37-0.42), a pooled specificity of 0.95 (95% CI: 0.93-0.96), a pooled positive-likelihood ratio (PLR) of 8.65 (95% CI, 4.47-16.73), and a pooled negative-likelihood ratio (NLR) of 0.41 (95% CI, 0.30-0.55), a diagnostic odds ratio (DOR) of 26.86 (95% CI: 15.73-45.89), and an area under the curve (AUC) of 0.9115. In conclusion, our study established that *WIF1* hypermethylation might be a promising diagnostic biomarker for CRC.

## INTRODUCTION

Colorectal cancer (CRC) is a complex multifactorial disease with an annual incidence of 1.2 million new cases and 600,000 deaths, ranking the third most frequent malignancy worldwide [1]. Although early screening and treatment reduces the morbidity and mortality [2–4], CRC remains the major cause of cancer death [5]. CRC often occurs in people older than 50 years, and its incidence is higher in men than women [6]. More than one-third of the deaths occur in patients aged greater than or equal to 80 years, and the rates of incidence and death are highest in blacks and lowest in Asians/Pacific Islanders [7].

As a common epigenetic modification, DNA methylation is modulated by both endogenous and exogenous factors [8, 9], including age, gender, ethnicity, and etc. DNA methylation often occurs in CpG island within or near gene promoter region [10]. Besides, aberrant DNA methylation is the main mechanism of gene inactivation in CRC patients [11, 12]. Currently, accumulating studies had identified a number of aberrant DNA methylation genes in tissue, serum, and stool DNA of CRC patients [13–16].

The Wnt signaling pathway is a highly conserved pathway that includes canonical Wnt pathway (Wnt/β-catenin pathway), planar cell polarity pathway and Wnt/Ca^2+^ pathway, [17, 18]. Aberrant Wnt pathway signaling is an early progression event of tumor and it occurs in 90% of CRC [19]. As a common antagonist of Wnt/β-catenin signaling, the main function of Wnt inhibitory factor 1 (*WIF1*) is to bind the extracellular Wnt ligands, disturbing β-catenin degradation, therefore inhibiting the Wnt/β-catenin pathway [20].

*WIF1* methylation has been widely studied in CRC, colorectal adenoma and normal adjacent tissue samples [21, 22]. However, the characterization of *WIF1* methylation in the diagnosis of CRC was still debatable. Studies with a small number of samples and different assay methylation methods might produce spurious results. In the present study, we performed a meta-analysis to evaluate *WIF1* methylation as a diagnostic biomarker for CRC.

## RESULTS

### Study characteristics and quality assessment

**Figure 1:**
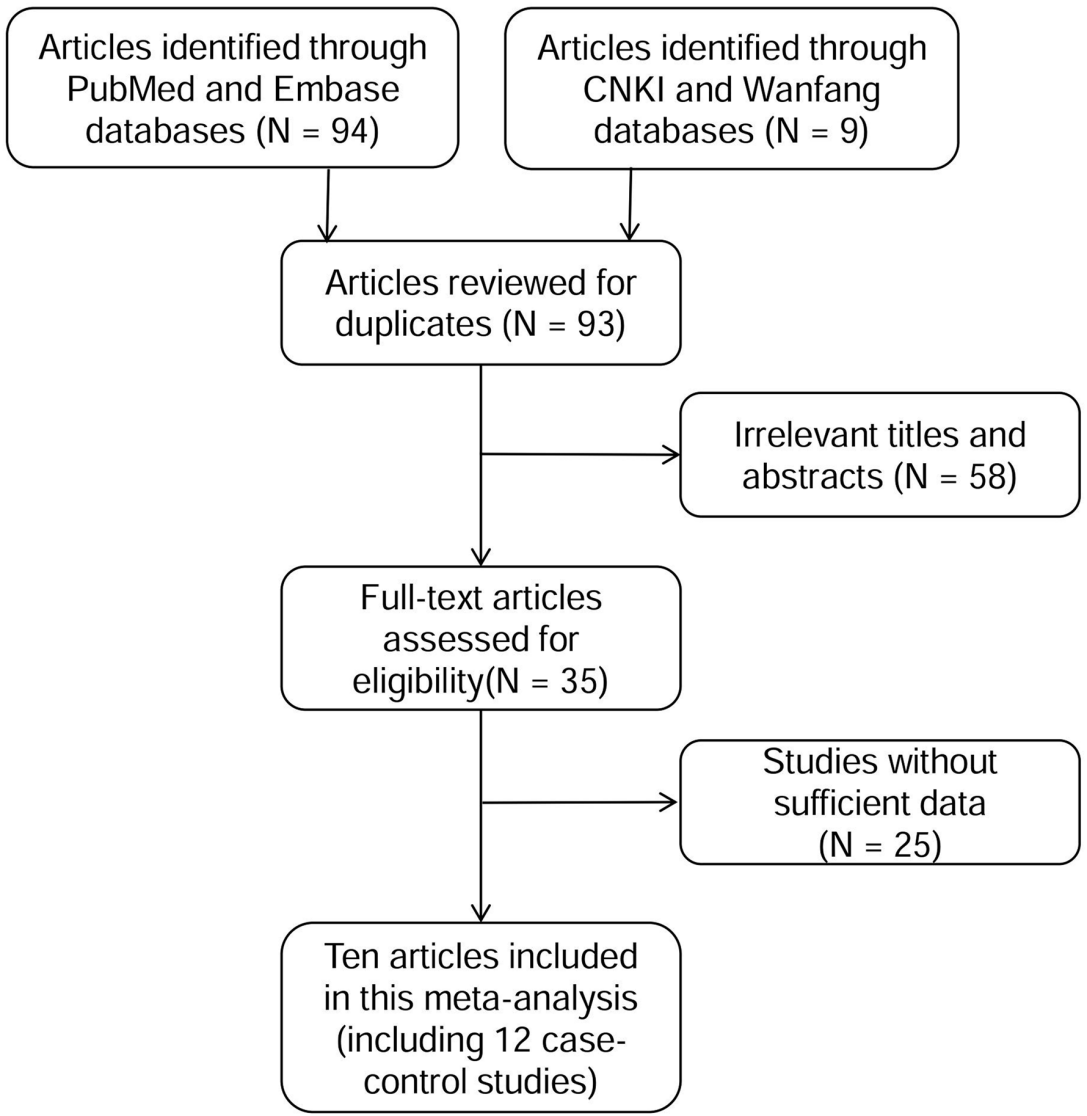
Flow diagram of the stepwise selection from relevant studies

As shown in Figure 1, a total of 103 articles were obtained for initial evaluation from PubMed, Embase, CNKI and Wanfang databases, and 93 articles remained after removing the duplicate literatures. A further check excluded 58 irrelevant articles and 25 articles without sufficient data. Ultimately, 10 eligible articles were enrolled in the current meta-analyses, which were involved with 12 case-control studies. All the eligible articles were published in English or Chinese. The information for *WIF1* methylation was collected from eligible studies and was shown in Table 1.

**Table 1:** The main characteristics of all available studies

First author	Year	Ethnicity	Samples	CRC		Normal	
				M+	Total	M+	Total
Qi Jian	2007	Asian	Tissues	61	72	9	58
Lee BB	2009	Asian	Tissues	180	243	3	148
Gao Bo	2010	Asian	Stool	19	27	0	8
Fang Yuan	2014	Asian	Tissues	13	14	2	16
Rania AD	2014	African	Tissues	73	83	17	43
Amiot	2014	European	Stool	18	247	1	157
Amiot	2014	European	Serum	31	247	2	157
Amiot	2014	European	Urine	26	247	2	157
Samaei NM	2014	Asian	Tissues	52	125	0	125
Hu Zhang	2014	Asian	Stool	29	48	1	30
Árpád V. Patai	2015	European	Tissues	14	17	2	15
Guangyue Yin	2016	Asian	Stool	30	50	2	32

M+: the number of methylation; total: the number of case or control.

The Quality Assessment of Diagnostic Accuracy Studies (QUADAS) assessment tool was used to assess the quality of the 10 studies. As shown in Supplementary Table 1, 8 out of the 10 studies did not mention the criteria that the index test results should be interpreted without knowledge of the results of the reference standard (item 10). And 3 out of 10 studies did not give the information to ensure if the time period between reference standard and index test was short enough to be reasonably sure that the target condition did not change between the two tests (item 4).

### Meta-analysis of *WIF1* methylation in CRC samples

**Figure 2:**
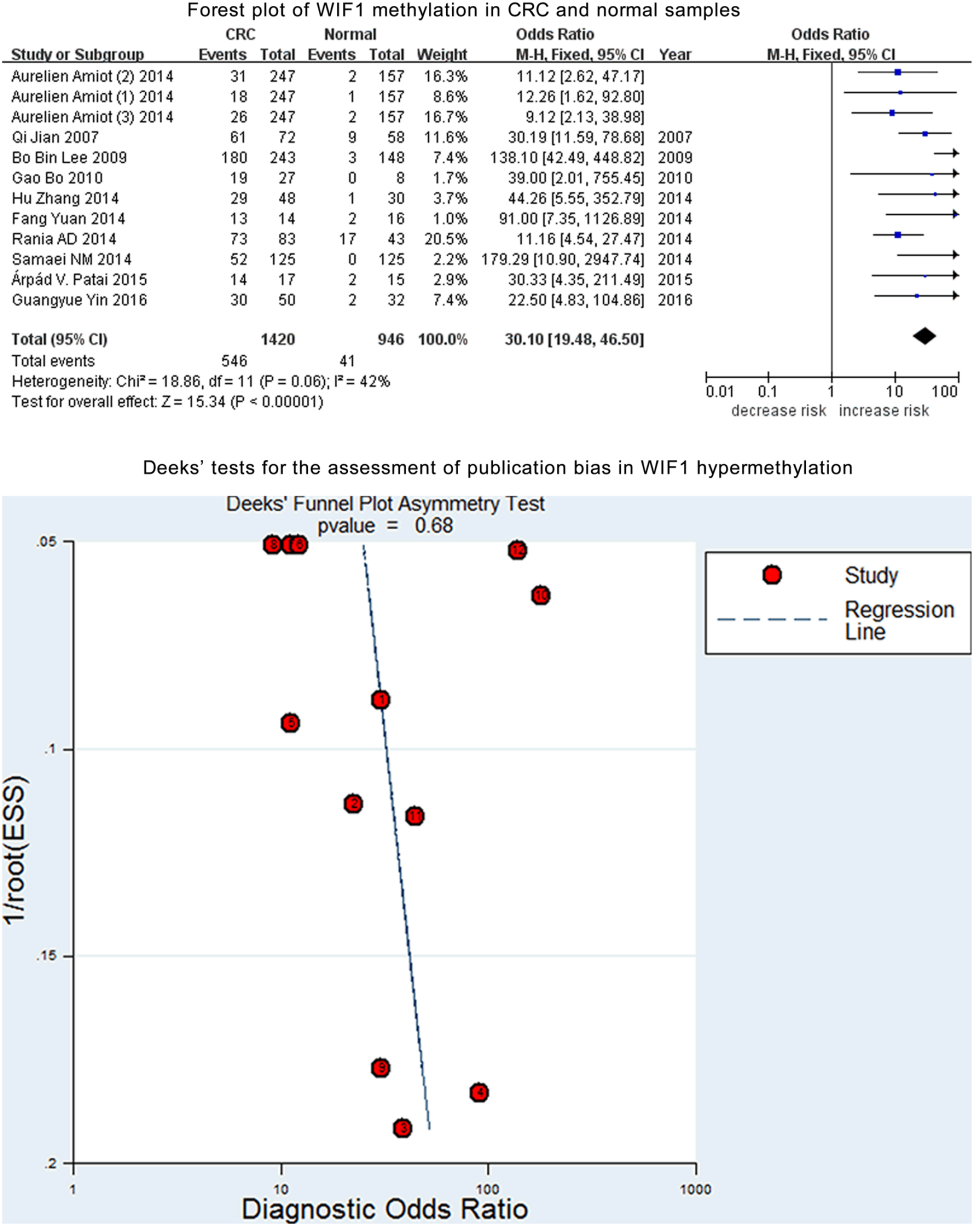
Forest plot of *WIF1* methylation in CRC and normal samples Deeks’ tests for the assessment of publication bias in WIF1 hypermethylation.

*WIF1* methylation was assessed among a total of 1420 CRC and 946 control samples from 12 case-control studies. Further analysis indicated a moderate heterogeneity in the current meta-analysis (*I^2^* = 42%). Therefore, a fixed-effect model was applied for the current meta-analysis. And our results showed a significant association of *WIF1* hypermethylation with CRC (OR = 30.10, 95% CI = 19.48-46.50, *P* < 0.001, Figure 2). Deeks’ funnel plot showed no publication bias in our meta-analysis (*P* = 0.68, Figure 2).

### Subgroup meta-analysis in CRC samples

Among the 12 studies, there were 7 Asian, 4 European and 1 African studies. Moreover, *WIF1* methylation was detected in CRC tissues (n = 6), feces (n = 5), and serum (n = 1), Therefore, we further performed subgroup meta-analysis by ethnicity and sample type. Our results showed a significant association of *WIF1* methylation with CRC risk in Asians, Europeans and Africans (Asians: 579 cases versus 417 controls, *P* < 0.001, OR = 64.33, 95% CI = 35.34-117.09, Europeans: 758 cases versus 486 controls, *P* < 0.001, OR = 11.83, 95% CI = 5.06-27.64, Africans: 83 cases versus 43 controls, *P* < 0.001, OR = 11.16, 95% CI = 4.54-27.47, Supplementary Figure 1). In addition, *WIF1* methylation was associated with CRC risk regardless of the tissue-based studies (*P* < 0.001, OR = 43.45, 95% CI = 15.38-122.73, Supplementary Figure 2) and feces-based and serum-based studies (*P* < 0.001, OR = 15.81, 95% CI = 7.74-32.26, Supplementary Figure 2). Our results also showed that the tissues-based studies had a larger heterogeneity (*I^2^* = 67%) than the feces-based and the serum-based studies (*I^2^* = 0%), suggesting that tissues-based subgroup was the main source of heterogeneity. Besides, subgroup meta-analysis by gender indicated no significant difference of *WIF1* methylation between females and males (*P* = 0.97). Subgroup meta-analysis by age showed that no significant difference of *WIF1* methylation was found between CRC patients aged greater than or equal to 60 years and CRC patients aged lower than 60 years (*P* = 0.66).

### *WIF1* methylation as a diagnostic biomarker for CRC

**Figure 3:**
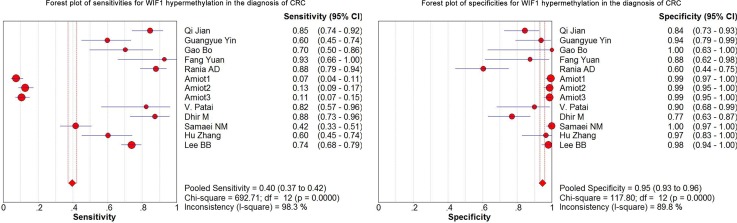
Forest plots of sensitivities and specificities for *WIF1* hypermethylation in the diagnosis of CRC

**Figure 4:**
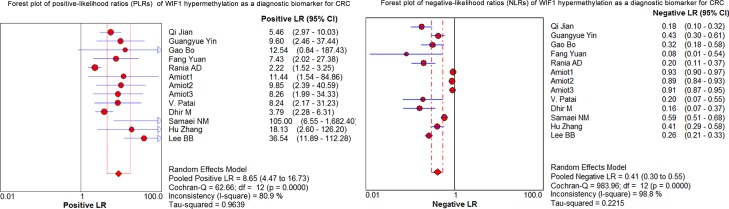
Forest plots of positive-likelihood ratios (PLRs) and negative-likelihood ratios (NLRs) of *WIF1* hypermethylation as a diagnostic biomarker for CRC

**Figure 5:**
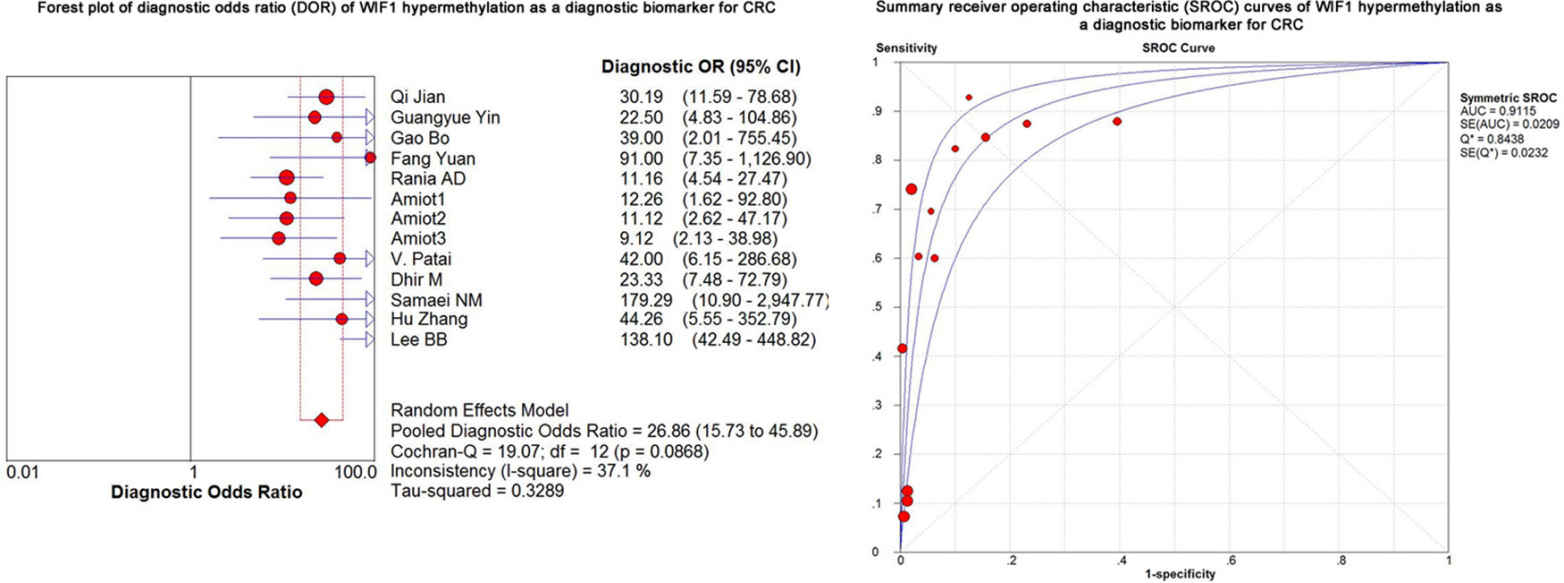
Forest plot of diagnostic odds ratio (DOR) and Summary receiver operating characteristic (SROC) curves of *WIF1* hypermethylation as a diagnostic biomarker for CRC

As shown in the preceding paragraphs, *WIF1* hypermethylation was more often seen in the CRC samples than the control samples. Thus, we estimated the diagnostic value of *WIF1* methylation in CRC. Our results showed there was a pooled sensitivity of 0.40 (95% CI: 0.37-0.42) and a pooled specificity of 0.95 (95% CI: 0.93-0.96) using *WIF1* methylation in the prediction of CRC risk (Figure 3). The positive-likelihood ratio (PLR) and the negative-likelihood ratio (NLR) of *WIF1* hypermethylation were more clinically valuable parameters compared to the specificity and the sensitivity [23]. In the present study, the pooled PLR was 8.65 (95% CI, 4.47-16.73), and the pooled NLR was 0.41 (95% CI, 0.30-0.55; Figure 4). As shown in Figure 5, *WIF1* hypermethylation could be used as a good diagnostic biomarker for CRC [diagnostic odds ratio (DOR) = 26.86 (15.73-45.89), area under the curve (AUC) = 0.9115].

Subsequently, we estimated the diagnostic values of *WIF1* methylation in CRC tissues and feces, respectively. There was a sensitivity of 0.71 (95% CI: 0.67-0.75), a specificity of 0.92 (95% CI: 0.89-0.94), a DOR of 45.46 and an AUC of 0.91 using CRC tissues to detect *WIF1* methylation (Supplementary Figure 3). And there was a sensitivity of 0.20 (95% CI: 0.17-0.23), a specificity of 0.98 (95% CI: 0.97-0.99), a DOR of 17.70, and an AUC of 0.94 using *WIF1* methylation as a diagnostic biomarker for CRC in feces (Supplementary Figure 4).

### Data mining study

**Figure 6:**
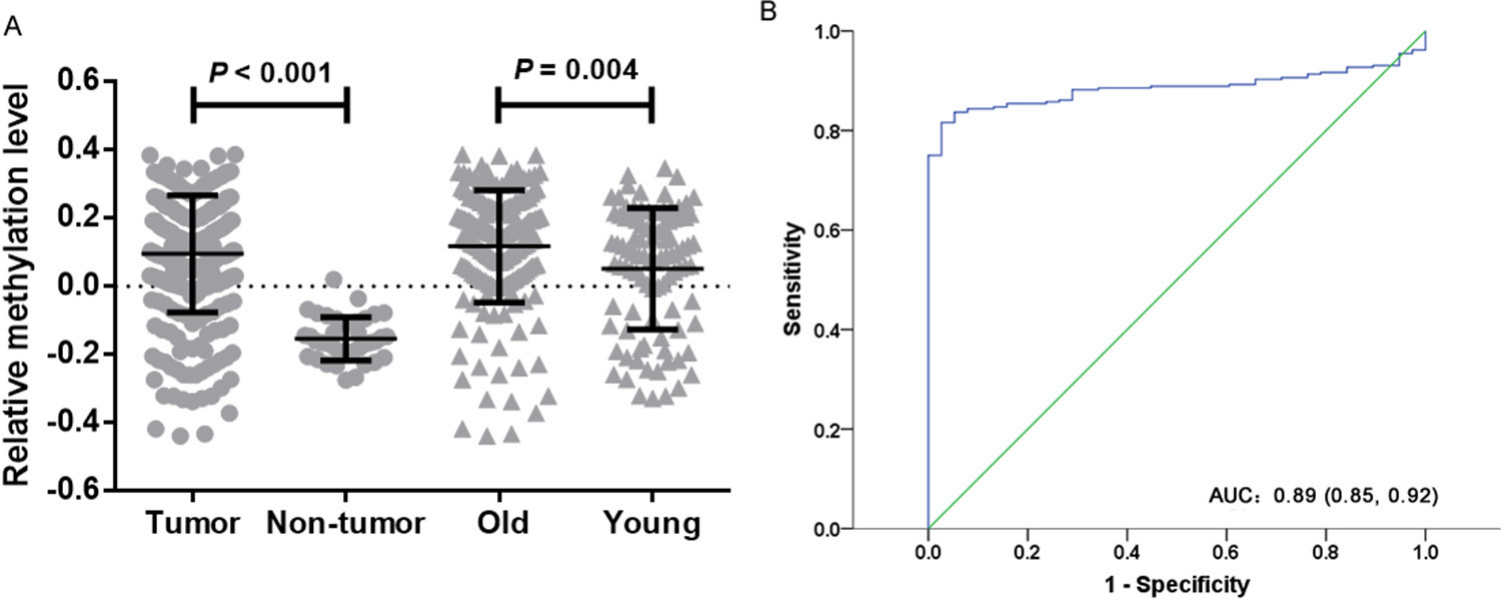
TCGA data analysis of *WIF1* methylation **(A)** Comparisons of *WIF1* methylation levels between tumor tissues and non-tumor tissues in CRC patients. Old stands for CRC patients aged greater than or equal to 60 years. Young stands for CRC patients aged lower than 60 years. Statistical values and the bar are presented as median with interquartile range. **(B)** The diagnostic value of *WIF1* methylation for CRC.

The Cancer Genome Atlas (TCGA) database also validated *WIF1* methylation in tumor tissues was significantly higher than that in non-tumor adjacent tissues. [median of mean β value (quartile range): 0.126 (0.018, 0.212) versus −0.164 (−0.197, −0.104), *P* < 0.001, Figure 6A]. And *WIF1* hypermethylation yielded an area under the curve (AUC) of 0.885 (95% CI: 0.850-0.920) with a sensitivity of 0.82, a specificity of 0.97 (Figure 6B). Besides, we examined the correlation between *WIF1* methylation and the clinicopathological features (gender and age) of CRC patients. Our results showed that *WIF1* were more frequently hypermethylated in the patients aged older than or equal to 60 years than those younger than 60 years [median of mean β value (quartile range): 0.143 (0.040, 0.228) versus 0.090 (-0.092, 0.200), *P* = 0.004, Figure 6A]. However, *WIF1* hypermethylation was not associated with gender (*P* = 0.17). Moreover, *WIF1* methylation was inversely correlated with gene expression in CRC (cg03509412, r = −0.326, *P* < 0.001, cg19427610, r = −0.322, *P* < 0.001, cg24166864, r = −0.301, *P* < 0.001, cg21383810, r = −0.033, *P* > 0.05).

Using the data of Gene Expression Omnibus (GEO) database (GSE32323), we found that *WIF1* expression in CRC cell lines (HCT116 and PKO) had an increasing tendency after 5’-AZA-deoxycytidine treatment (5-AZA) (Supplementary Figure 5, Fold change > 1.10). Therefore, *WIF1* were likely to be hypermethylated in CRC cell lines, which potentially suppressed *WIF1* expression.

## DISCUSSION

Dysregulation of Wnt signaling pathway genes (including *WIF1*) often results in uncontrolled β-catenin signaling and excessive proliferation that predisposes cells to tumorigenesis [24]. *WIF1* is a common target of epigenetic silencing in various human cancers [25]. Since numerous studies had found *WIF1* hypermethylation in CRC, we performed a meta-analysis to evaluate *WIF1* methylation as a diagnostic biomarker for CRC.

In the present meta-analysis, the DOR value was 26.86, suggesting that CRC patients were more likely to be diagnosed as positive by *WIF1* methylation test. To note, DOR as an overall measure of diagnostic accuracy was often computed as the odds of positivity among patients [26]. Compared to guaiac fecal occult blood tests, the detection of *WIF1* methylation in either serum or urine has a higher accuracy for advanced colorectal neoplasia [27]. As one of the most widely used tumor biomarkers worldwide, serum carcino-embryonic antigen (CEA) was used to screen for CRC [28]. However, the sensitivity and the specificity of CEA were not high in screening for CRC (sensitivity = 0.36; specificity = 0.87) [29]. Moreover, the sensitivities of CEA for detecting CRC up to 1 and 4 years before clinical presentation were only 0.25 and 0.13, respectively [30]. Our study showed that *WIF1* hypermethylation yielded a high AUC of 0.9115 (sensitivity: 0.40; specificity: 0.95) in CRC. Similarly, TCGA data also showed a high diagnostic performance of *WIF1* hypermethylation for CRC (AUC = 0.89, sensitivity = 0.82; specificity = 0.97). Future study is needed to check the joint diagnostic value of *WIF1* hypermethylation and conventional plasma proteins for CRC.

Although the specificity and AUC were satisfied, the sensitivity of *WIF1* hypermethylation was moderate (pooled sensitivity = 0.40). The sensitivity of *WIF1* methylation in feces and serum (sensitivity = 0.20) was lower than that in tissues (sensitivity = 0.71). Fragmentation of the cell-free DNA and the low amount of genomic DNA in feces or serum might be responsible for the reduced sensitivity in the methylation assay [31]. A recent study identified a sensitivity of 0.87 and a specificity of 0.92 (AUC = 0.927) using the methylation of *APC*, *MGMT*, *RASSF2A*, and *WIF1* as a diagnostic biomarker panel for CRC [31]. Thus, it is necessary to combine with other gene methylation or protein biomarkers to improve the sensitivity of *WIF1* methylation as a noninvasive biomarker for CRC.

*WIF1* promoter hypermethylation was shown to down regulate the *WIF1* expression [32–34]. Analysis of TCGA data showed that *WIF1* methylation was inversely correlated with gene expression in CRC. GEO data showed *WIF1* expression in CRC cell lines increased over 1.1 fold after demethylation treatment (GSE32323). The above evidence suggested that *WIF1* hypermethylation might contribute to the risk of CRC by its down-regulation of *WIF1* expression.

Cancer incidence and mortality vary a lot between and within racial and ethnic groups. The incidence and mortality from CRC was higher among blacks when compared with other race-ethnicities [35]. Racial disparities may cause the mixed results of DNA methylation in CRC. Our subgroup meta-analysis by ethnicity suggested the significant association of *WIF1* methylation with CRC existed in Asians, Europeans and Africans. However, due to the small amount of studies, more studies should be performed in Europeans and Africans to confirm this observation.

Gender differences in the incidence of CRC have been observed in Asia and North America [36–38]. In addition, the incidence of CRC is low at ages younger than 50 years, but increases with age [6]. Our meta-analysis and analysis of TCGA data showed no significant difference of *WIF1* methylation between males and females. Analysis of TCGA data showed that *WIF1* were more frequently hypermethylated in CRC patients aged greater than or equal to 60 years. However, there was no significant difference in *WIF1* methylation between age subgroups in our meta-analysis. This discrepancy might be due to a paucity of age information in the meta-analysis. Future studies are needed to explore the potential relationship of *WIF1* methylation with the incidence age and the gender status of CRC.

There are the following aspects of main limitations in our meta-analysis to be noted. Firstly, selection bias is inevitable due to the strategy restricted to articles published in English and Chinese. Secondly, we did not study the methylation status in histological subtypes or different clinical stages due to the insufficient information. Thirdly, the regions for methylation detection were not taken into consideration in our meta-analysis. Therefore, a further study is needed to distinguish the different regions to confirm which specific region could represent *WIF1* methylation.

In conclusion, our meta-analysis established *WIF1* hypermethylation had a potential in the clinical diagnosis for CRC.

## MATERIALS AND METHODS

### Literature selection

We performed a systemic search using two relevant literature databases (PubMed and Embase) and two Chinese databases (CNKI and Wanfang). Eligible literatures updated until August of 2017 were identified using “(Wnt inhibitory factor 1 or *WIF1*) and (colorectal cancer or colorectal tumor or colorectal carcinoma or colorectal neoplasm) and (methylation or epigene^*^)” as the keywords. Studies included in the meta-analysis were required to meet all the following criteria: [1] the study should have full text to be confirmed as an original study on the association between CRC and *WIF1* methylation; [2] the study should contain case-control cohorts or have subgroups according to ethnicity, gender or age; [3] control samples must be non-cancerous ones from healthy persons or the adjacent non-cancerous tissues of CRC patients; [4] the study had sufficient data to calculate true positive, false positive, true negative and false negative; [5] when the same set of patients reported more than once, only the most complete one was included to avoid data overlapping.

### Data extraction and quality assessment

For all eligible literatures, the following data was extracted respectively by three authors (HH, BL and CZ) with a standardized data extraction form which contained gene name, the first author's name, year of publication, patient ethnicity, size and type of sample, methylation status, and specific information about subgroups. Two authors (HH and BL) estimated the quality of 10 studies independently according to the QUADAS [39].

### Data mining study

We extracted the 450K array data of 288 colon adenocarcinoma samples and 38 non-tumor samples from TCGA website (https://tcga-data.nci.nih.gov/docs/publications/tcga/). And we calculated the average methylation level of four CpG sites (cg03509412, cg19427610, cg24166864, cg21383810) to validate the performance of *WIF1* methylation in CRC. *WIF1* mRNA expression was retrieved from GEO database (www.ncbi.nlm.nih.gov/geo, accession no. GSE32323). We focused on the expression changes of *WIF1* in two CRC cell lines (HCT116, PKO) with and without 5’-AZA-deoxycytidine treatment.

### Statistical analysis

Meta-analysis was performed using Review Manager 5, Meta-Disc 1.4 and Stata SE12.0 software. The ORs and 95% CIs were extracted or calculated to evaluate the strength of the association between *WIF1* methylation and CRC risk. Overall ORs and 95% CIs were calculated with the data in the selected studies. *I^2^* statistic was used to estimate the heterogeneity of the studies in the meta-analysis [40]. The random-effect model was applied for the meta-analysis when *I^2^* > 50%, otherwise, the fixed-effect model was used [41]. Subgroup analyses were also performed to explore the source of heterogeneity. For the diagnostic meta-analysis, we extracted true positive, false positive, true negative and false negative from each eligible study. To estimate the diagnostic performance of *WIF1* methylation, we calculated forest plots of sensitivity, specificity, the PLR, NLR, and DOR, and we also drew the summary receiver operator characteristic (SROC) curve [23]. Deeks’ funnel plot asymmetry test was performed to assess the potential publication bias [42]. A nonparametric Mann-Whitney U test was used to assess the methylation differences between tumor tissues and normal tissues from TCGA database. Receiver operating characteristic (ROC) analysis showed the diagnostic value of *WIF1* methylation in TCGA database. Differences were considered statistically significant if *P* < 0.05.

## SUPPLEMENTARY MATERIALS FIGURES AND TABLES



## References

[1] Chen D, Huang JF, Liu K, Zhang LQ, Yang Z, Chuai ZR, Wang YX, Shi DC, Huang Q, Fu WL (2014). BRAFV600E mutation and its association with clinicopathological features of colorectal cancer: a systematic review and meta-analysis. PLoS One.

[2] U.S. Preventive Services Task Force (2008). Screening for colorectal cancer: U.S. Preventive Services Task Force recommendation statement. Ann Intern Med.

[3] Zauber AG, Lansdorp-Vogelaar I, Knudsen AB, Wilschut J, van Ballegooijen M, Kuntz KM (2008). Evaluating test strategies for colorectal cancer screening: a decision analysis for the U.S. Preventive Services Task Force. Ann Intern Med.

[4] Holme O, Loberg M, Kalager M, Bretthauer M, Hernan MA, Aas E, Eide TJ, Skovlund E, Schneede J, Tveit KM, Hoff G (2014). Effect of flexible sigmoidoscopy screening on colorectal cancer incidence and mortality: a randomized clinical trial. JAMA.

[5] Ferlay J, Shin HR, Bray F, Forman D, Mathers C, Parkin DM (2010). Estimates of worldwide burden of cancer in 2008: GLOBOCAN 2008. Int J Cancer.

[6] Brenner H, Kloor M, Pox CP (2014). Colorectal cancer. Lancet.

[7] Siegel R, Desantis C, Jemal A (2014). Colorectal cancer statistics, 2014. CA Cancer J Clin.

[8] Jiang W, Wang PG, Zhan Y, Zhang D (2014). Prognostic value of p16 promoter hypermethylation in colorectal cancer: a meta-analysis. Cancer Invest.

[9] Li X, Yao X, Wang Y, Hu F, Wang F, Jiang L, Liu Y, Wang D, Sun G, Zhao Y (2013). MLH1 promoter methylation frequency in colorectal cancer patients and related clinicopathological and molecular features. PLoS One.

[10] Coppede F (2014). Epigenetic biomarkers of colorectal cancer: focus on DNA methylation. Cancer Lett.

[11] Issa JP (2004). CpG island methylator phenotype in cancer. Nat Rev Cancer.

[12] Kondo Y, Issa JP (2004). Epigenetic changes in colorectal cancer. Cancer Metastasis Rev.

[13] Cheng YW, Pincas H, Huang J, Zachariah E, Zeng Z, Notterman DA, Paty P, Barany F (2014). High incidence of LRAT promoter hypermethylation in colorectal cancer correlates with tumor stage. Med Oncol.

[14] Yuan Y, Qu B, Yan J, Wang H, Yin L, Han Q (2015). Diagnostic value of aberrant gene methylation in stool samples for colorectal cancer or adenomas: a meta-analysis. Panminerva Med.

[15] Perez-Carbonell L, Balaguer F, Toiyama Y, Egoavil C, Rojas E, Guarinos C, Andreu M, Llor X, Castells A, Jover R, Boland CR, Goel A (2014). IGFBP3 methylation is a novel diagnostic and predictive biomarker in colorectal cancer. PLoS One.

[16] Tham C, Chew M, Soong R, Lim J, Ang M, Tang C, Zhao Y, Ong SY, Liu Y (2014). Postoperative serum methylation levels of TAC1 and SEPT9 are independent predictors of recurrence and survival of patients with colorectal cancer. Cancer.

[17] Galamb O, Kalmar A, Peterfia B, Csabai I, Bodor A, Ribli D, Krenács T, ÁV Patai, Wichmann B, Barták BK, Tóth K, Valcz G, Spisák S (2016). Aberrant DNA methylation of WNT pathway genes in the development and progression of CIMP-negative colorectal cancer. Epigenetics.

[18] Novellasdemunt L, Antas P, Li VS (2015). Targeting Wnt signaling in colorectal cancer. A review in the theme: cell signaling: proteins, pathways and mechanisms. Am J Physiol Cell Physiol.

[19] Fodde R, Smits R, Clevers H (2001). APC, signal transduction and genetic instability in colorectal cancer. Nat Rev Cancer.

[20] Liang J, Zhou H, Peng Y, Xie X, Li R, Liu Y, Xie Q, Lin Z (2016). β-catenin expression negatively correlates with WIF1 and predicts poor clinical outcomes in patients with cervical cancer. Biomed Res Int.

[21] Paluszczak J, Hemmerling D, Kostrzewska-Poczekaj M, Jarmuz-Szymczak M, Grenman R, Wierzbicka M, Baer-Dubowska W (2014). Frequent hypermethylation of WNT pathway genes in laryngeal squamous cell carcinomas. J Oral Pathol Med.

[22] Samaei NM, Yazdani Y, Alizadeh-Navaei R, Azadeh H, Farazmandfar T (2014). Promoter methylation analysis of WNT/beta-catenin pathway regulators and its association with expression of DNMT1 enzyme in colorectal cancer. J Biomed Sci.

[23] Yu W, Wang Z, Shen LI, Wei Q (2016). Circulating microRNA-21 as a potential diagnostic marker for colorectal cancer: a meta-analysis. Mol Clin Oncol.

[24] Delmas AL, Riggs BM, Pardo CE, Dyer LM, Darst RP, Izumchenko EG, Monroe M, Hakam A, Kladde MP, Siegel EM, Brown KD (2011). WIF1 is a frequent target for epigenetic silencing in squamous cell carcinoma of the cervix. Carcinogenesis.

[25] Ying Y, Tao Q (2009). Epigenetic disruption of the WNT/beta-catenin signaling pathway in human cancers. Epigenetics.

[26] Westwood ME, Whiting PF, Kleijnen J (2005). How does study quality affect the results of a diagnostic meta-analysis?. BMC Med Res Methodol.

[27] Amiot A, Mansour H, Baumgaertner I, Delchier JC, Tournigand C, Furet JP, Carrau JP, Canoui-Poitrine F, I; Sobhani (2014). CRC group of Val De Marne. The detection of the methylated Wif-1 gene is more accurate than a fecal occult blood test for colorectal cancer screening. PLoS One.

[28] Du M, Liu S, Gu D, Wang Q, Zhu L, Kang M, Shi D, Chu H, Tong N, Chen J, Adams TS, Zhang Z, Wang M (2014). Clinical potential role of circulating microRNAs in early diagnosis of colorectal cancer patients. Carcinogenesis.

[29] Fletcher RH (1986). Carcinoembryonic antigen. Ann Intern Med.

[30] Thomas DS, Fourkala EO, Apostolidou S, Gunu R, Ryan A, Jacobs I, Menon U, Alderton W, Gentry-Maharaj A, Timms JF (2015). Evaluation of serum CEA, CYFRA21-1 and CA125 for the early detection of colorectal cancer using longitudinal preclinical samples. Br J Cancer.

[31] Lee BB, Lee EJ, Jung EH, Chun HK, Chang DK, Song SY, Park J, Kim DH (2009). Aberrant methylation of APC, MGMT, RASSF2A, and Wif-1 genes in plasma as a biomarker for early detection of colorectal cancer. Clin Cancer Res.

[32] Abdelmaksoud-Dammak R, Miladi-Abdennadher I, Saadallah-Kallel A, Khabir A, Sellami-Boudawara T, Frikha M, Daoud J, Mokdad-Gargouri R (2014). Downregulation of WIF-1 and Wnt5a in patients with colorectal carcinoma: clinical significance. Tumour Biol.

[33] Lin B, Hong H, Jiang X, Li C, Zhu S, Tang N, Wang X, She F, Chen Y (2017). WNT inhibitory factor 1 promoter hypermethylation is an early event during gallbladder cancer tumorigenesis that predicts poor survival. Gene.

[34] Wang N, Wang Z, Wang Y, Xie X, Shen J, Peng C, You J, Peng F, Tang H, Guan X, Chen J (2015). Dietary compound isoliquiritigenin prevents mammary carcinogenesis by inhibiting breast cancer stem cells through WIF1 demethylation. Oncotarget.

[35] Siegel RL, Miller KD, Jemal A (2016). Cancer statistics, 2016. CA Cancer J Clin.

[36] Chou CL, Weng SF, Lin JK, Chang SC (2013). Role for gender in colorectal cancer risk: a Taiwan population-based study. Int J Colorectal Dis.

[37] de Kok IM, Wong CS, Chia KS, Sim X, Tan CS, Kiemeney LA, Verkooijen HM (2008). Gender differences in the trend of colorectal cancer incidence in Singapore, 1968-2002. Int J Colorectal Dis.

[38] Woods SE, Basho S, Engel A (2006). The influence of gender on colorectal cancer stage: the state of Ohio, 1996-2001. J Womens Health (Larchmt).

[39] Whiting P, Rutjes AW, Reitsma JB, Bossuyt PM, Kleijnen J (2003). The development of QUADAS: a tool for the quality assessment of studies of diagnostic accuracy included in systematic reviews. BMC Med Res Methodol.

[40] Higgins JP, Thompson SG, Deeks JJ, Altman DG (2003). Measuring inconsistency in meta-analyses. BMJ.

[41] Bax L, Ikeda N, Fukui N, Yaju Y, Tsuruta H, Moons KG (2009). More than numbers: the power of graphs in meta-analysis. Am J Epidemiol.

[42] Deeks JJ, Macaskill P, Irwig L (2005). The performance of tests of publication bias and other sample size effects in systematic reviews of diagnostic test accuracy was assessed. J Clin Epidemiol.

