# Supplementary material for: Diagnostic value of WIF1 methylation for colorectal cancer: a meta-analysis
Source: Oncotarget. 2018 Jan 3;9(4):5378–86. doi: 10.18632/oncotarget.23870 (PMC5797056; doi:10.18632/oncotarget.23870)
Supplement: Supplementary file 1 [file oncotarget-09-5378-s001.pdf]

Diagnostic value of *WIF1* methylation for colorectal cancer: a meta-analysis

SUPPLEMENTARY MATERIALS

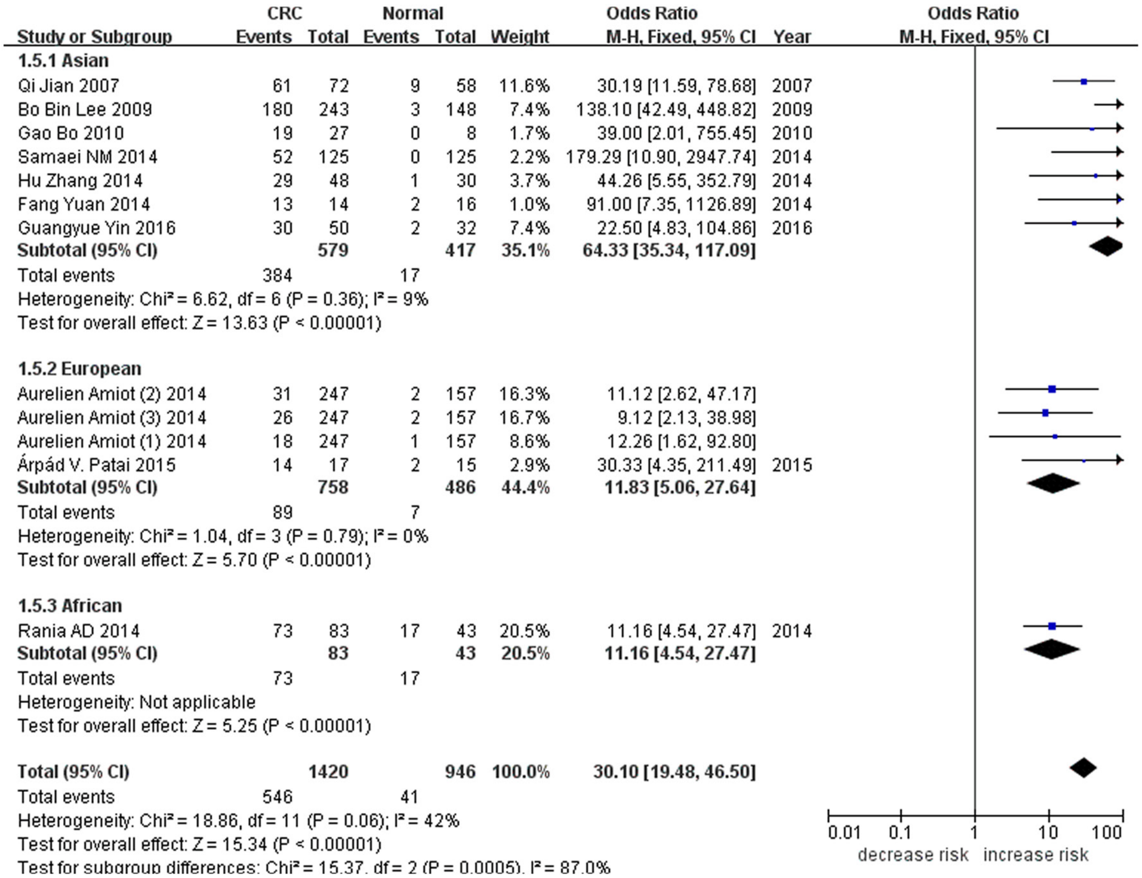

Supplementary Figure 1: Subgroup meta-analysis by ethnicity of *WIF1* methylation in CRC.

Study or Subgroup	CRC Events	CRC Total	Normal Events	Normal Total	Weight	Odds Ratio M-H, Random, 95% CI	Year
**1.4.1 Tissues**							
Qi Jian 2007	61	72	9	58	14.1%	30.19 [11.59, 78.68]	2007
Bo Bin Lee 2009	180	243	3	148	11.9%	138.10 [42.49, 448.82]	2009
Fang Yuan 2014	13	14	2	16	4.5%	91.00 [7.35, 1126.89]	2014
Samaei NM 2014	52	125	0	125	3.8%	179.29 [10.90, 2947.74]	2014
Rania AD 2014	73	83	17	43	14.8%	11.16 [4.54, 27.47]	2014
Árpád V. Patai 2015	14	17	2	15	6.7%	30.33 [4.35, 211.49]	2015
**Subtotal (95% CI)**		**554**		**405**	**55.8%**	**43.45 [15.38, 122.73]**	
Total events	393		33				
Heterogeneity: Tau² = 1.01; Chi² = 15.31, df = 5 (P = 0.009); I² = 67%							
Test for overall effect: Z = 7.12 (P < 0.00001)							
**1.4.2 Feces and Serum**							
Aurelien Amiot (3) 2014	26	247	2	157	9.6%	9.12 [2.13, 38.98]	
Aurelien Amiot (2) 2014	31	247	2	157	9.7%	11.12 [2.62, 47.17]	
Aurelien Amiot (1) 2014	18	247	1	157	6.3%	12.26 [1.62, 92.80]	
Gao Bo 2010	19	27	0	8	3.5%	39.00 [2.01, 755.45]	2010
Hu Zhang 2014	29	48	1	30	6.1%	44.26 [5.55, 352.79]	2014
Guangyue Yin 2016	30	50	2	32	9.0%	22.50 [4.83, 104.86]	2016
**Subtotal (95% CI)**		**866**		**541**	**44.2%**	**15.81 [7.74, 32.26]**	
Total events	153		8				
Heterogeneity: Tau² = 0.00; Chi² = 2.35, df = 5 (P = 0.80); I² = 0%							
Test for overall effect: Z = 7.58 (P < 0.00001)							
**Total (95% CI)**		**1420**		**946**	**100.0%**	**27.03 [14.83, 49.26]**	
Total events	546		41				
Heterogeneity: Tau² = 0.42; Chi² = 18.86, df = 11 (P = 0.06); I² = 42%							
Test for overall effect: Z = 10.77 (P < 0.00001)							
Test for subgroup differences: Chi² = 2.47, df = 1 (P = 0.12), I² = 59.6%							

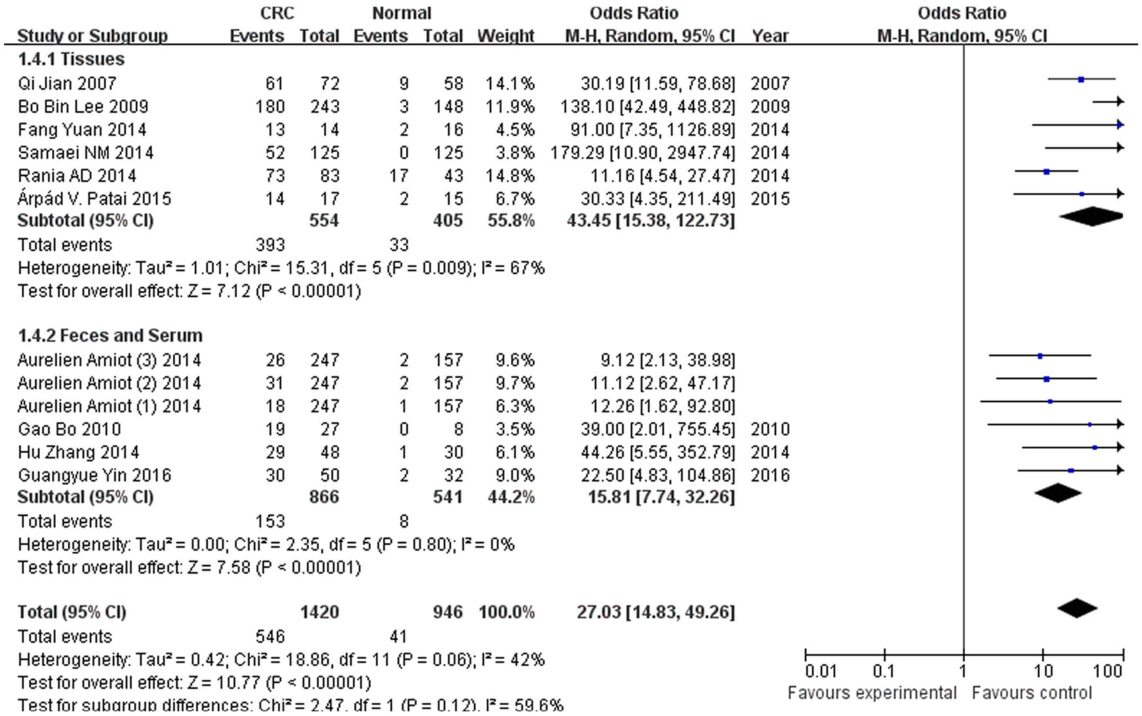

Supplementary Figure 2: Subgroup meta-analyses by sample type of *WIF1* methylation in CRC.

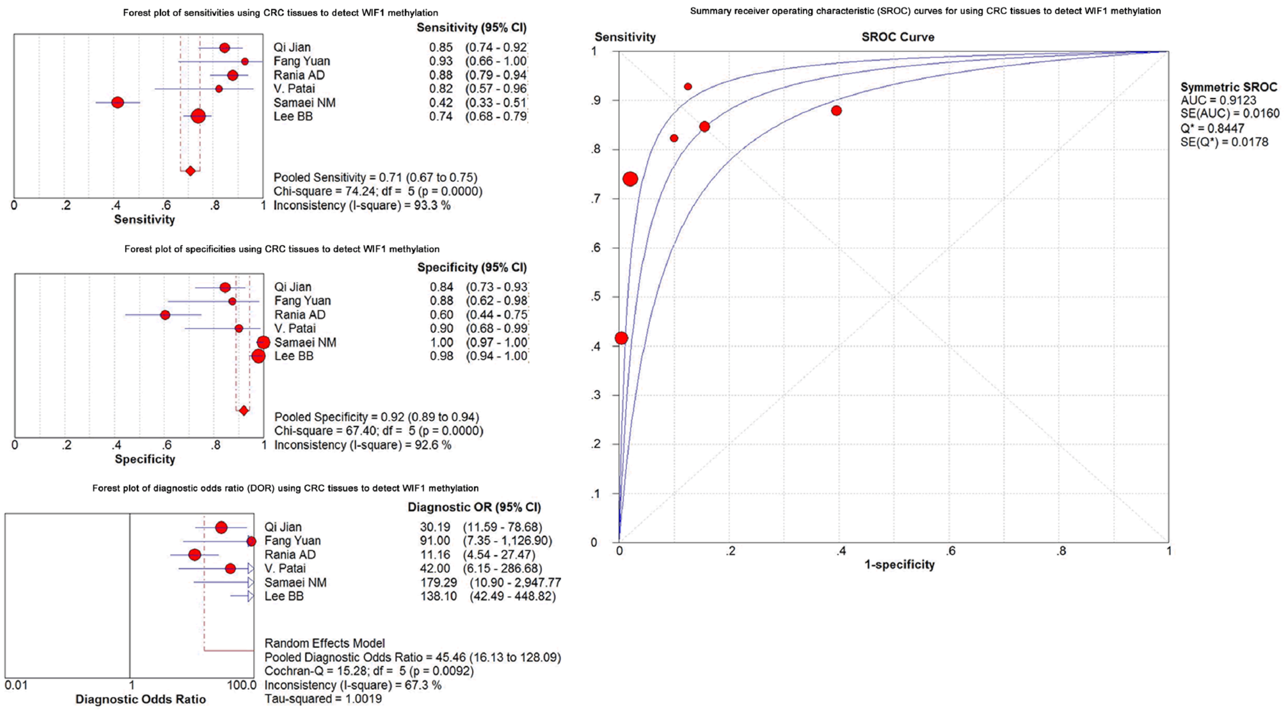

Supplementary Figure 3: Forest plots of sensitivities, specificities diagnostic odds ratio (DOR), and summary receiver operating characteristic (SROC) curves of *WIF1* hypermethylation as a diagnostic biomarker for CRC in tissues. AUC stands for area under the curve.

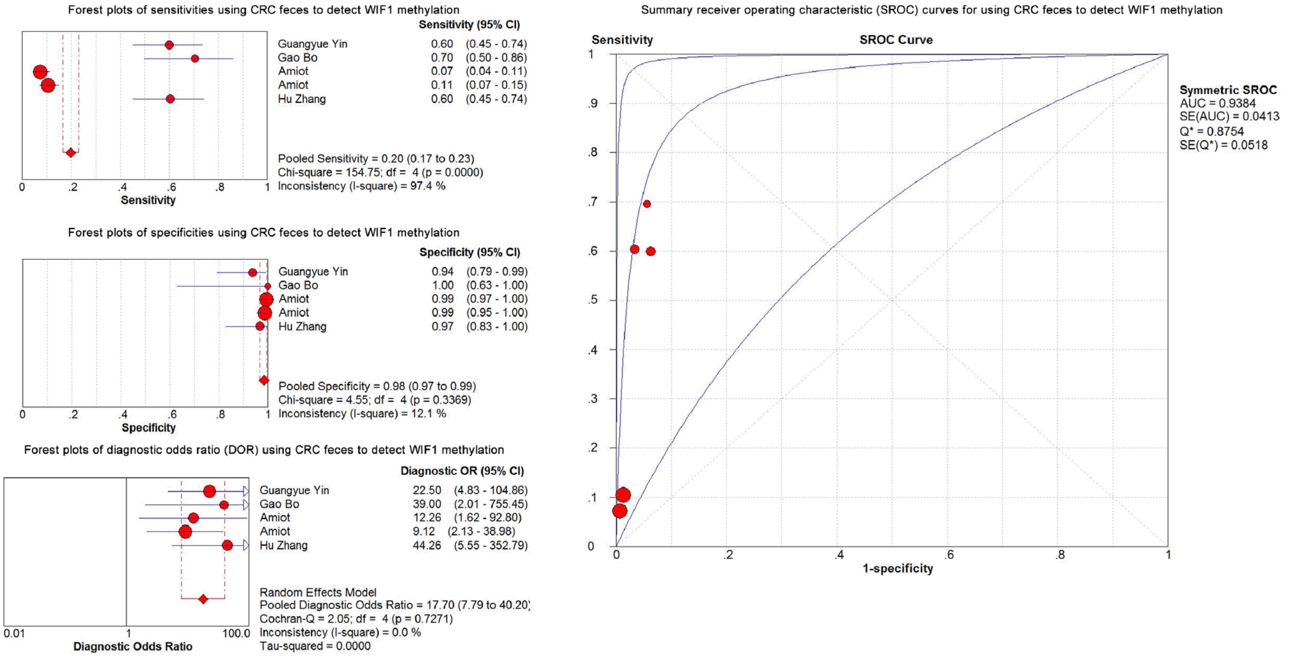

Supplementary Figure 4: Forest plots of sensitivities, specificities diagnostic odds ratio (DOR), and summary receiver operating characteristic (SROC) curves of *WIF1* hypermethylation as a diagnostic biomarker for CRC in feces. AUC stands for area under the curve.

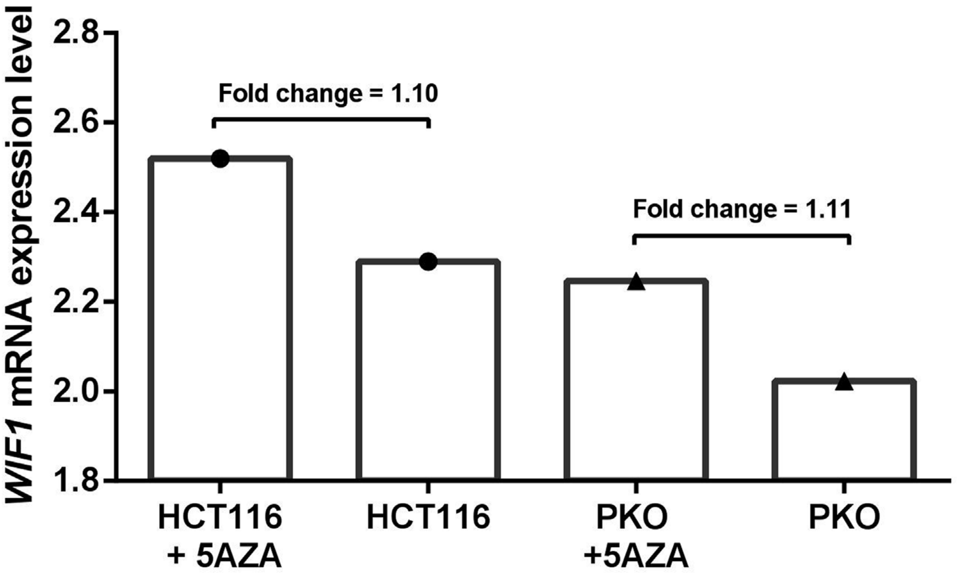

Supplementary Figure 5: The expression value changes with and without 5AZA treatment in CRC cell lines (HCT116 and PKO) derive from the GEO database (GSE32323). *WIF1* expression profiles for two CRC cell lines were measured by Affymetrix HG-U133 Plus 2.0 arrays. And the normalized gene expression levels were presented as log2-transformed values by robust multi-array average. 5AZA: cell line with 5-AZA-deoxycitidine treatment.

Supplementary Table 1: QUADAS assessment for the eligible studies

Item	Qi et al	Yin et al	Gao et al	Fang *et al*	Rania *et al*	Amiot *et al*	Árpád et al	Samaei *et al*	Hu et al	Lee et al
1. Was the spectrum of patients representative of the patients who will receive the test in practice?	Yes	Yes	Yes	Yes	Yes	Yes	Yes	Yes	Yes	Yes
2. Were selection criteria clearly described?	Yes	Yes	Yes	Yes	Yes	Yes	Yes	Yes	Yes	Yes
3. Is the reference standard likely to correctly classify the target condition?	Yes	Yes	Yes	Yes	Yes	Yes	Yes	Yes	Yes	Yes
4. Is the time period between reference standard and index test short enough to be reasonably sure that the target condition did not change between the two tests?	Yes	Not clear	Yes	Yes	Yes	Yes	Not clear	Yes	Yes	Yes
5. Did the whole sample or a random selection of the sample receive verifcation using a reference standard?	Yes	Yes	Yes	Yes	Yes	Yes	Yes	Yes	Yes	Yes
6. Did patients receive the same reference standard regardless of the index test result?	Yes	Yes	Yes	Yes	Yes	Yes	Yes	Yes	Yes	Yes
7. Was the reference standard independent of the index test (i.e., the index test did not form part of the reference standard)?	Yes	Yes	Yes	Yes	Yes	Yes	Yes	Yes	Yes	Yes
8. Was the execution of the index test described in suffcient detail to permit replication of the test?	Yes	Yes	Yes	Yes	Yes	Yes	Yes	Yes	Yes	Yes
9. Was the execution of the reference standard described in sufficient detail to permit its replication?	Yes	Yes	Yes	Yes	Yes	Yes	Yes	Yes	Yes	Yes
10. Were the index test results interpreted without knowledge of the results of the reference standard?	Not clear	Not clear	Yes	Not clear	Not clear	Not clear	Not clear	Not clear	Yes	Not clear
11. Were the reference standard results interpreted without knowledge of the results of the index test?	Yes	Yes	Yes	Yes	Yes	Yes	Yes	Yes	Yes	Yes
12. Were the same clinical data available when test results were interpreted as would be available when the test is used in practice?	Yes	Yes	Yes	Yes	Yes	Yes	Yes	Yes	Yes	Yes
13. Were uninterpretable/ intermediate test results reported?	Yes	Yes	Yes	Yes	Yes	Yes	Yes	Yes	Yes	Yes
14. Were withdrawals from the study explained?	Yes	Yes	Yes	Yes	Yes	Yes	Yes	Yes	Yes	Yes
